# Pathogen Risk Analysis for Wild Amphibian Populations Following the First Report of a Ranavirus Outbreak in Farmed American Bullfrogs (*Lithobates catesbeianus*) from Northern Mexico

**DOI:** 10.3390/v11010026

**Published:** 2019-01-03

**Authors:** Bernardo Saucedo, José M. Serrano, Mónica Jacinto-Maldonado, Rob S. E. W. Leuven, Abraham A. Rocha García, Adriana Méndez Bernal, Andrea Gröne, Steven J. van Beurden, César M. Escobedo-Bonilla

**Affiliations:** 1 Department of Pathobiology, Faculty of Veterinary Medicine, Utrecht University, 3584 CL Utrecht, The Netherlands; b.saucedogarnica@uu.nl (B.S.); a.groene@uu.nl (A.G.); steven.vanbeurden@gupta-strategists.nl (S.J.v.B.); 2 Laboratorio de Genética y Evolución, Departamento de Ciencias Ecológicas, Facultad de Ciencias, Universidad de Chile, Las Palmeras, Santiago 3425, Chile; jose.rano@gmail.com; 3 Programa de Fisiología y Biofísica, Facultad de Medicina, Universidad de Chile, Santiago 8380453, Chile; 4 Wildlife and Laboratory Animals, Department of Ethology, Faculty of Veterinary Medicine, National Autonomous University of Mexico, Mexico City 045010, Mexico; monica.jacinto@c3.unam.mx; 5 C3-Complexity sciences Center, Autonomous University of Mexico, Mexico City 045010, Mexico; 6 Department of Animal Ecology and Physiology, Institute for Water and Wetland Research, Radboud University, 6500 GL Nijmegen, The Netherlands; r.leuven@science.ru.nl; 7 Netherlands Centre of Expertise on Exotic Species, 6500 GL Nijmegen, The Netherlands; 8 Department of Pathology 04510, Faculty of Veterinary Medicine, National Autonomous University of Mexico, Mexico City 045010, Mexico; ab_abraham88@hotmail.com (A.A.R.G.); mvzadrimb@gmail.com (A.M.B.); 9 Department of Aquaculture, Instituto Politécnico, Nacional-CIIDIR Unidad Sinaloa, Guasave Sinaloa 81101, Mexico

**Keywords:** amphibians, histopathology, immunohistochemistry, Mexico, outbreak, ranavirus, risk assessment

## Abstract

Ranaviruses are the second deadliest pathogens for amphibian populations throughout the world. Despite their wide distribution in America, these viruses have never been reported in Mexico, the country with the fifth highest amphibian diversity in the world. This paper is the first to address an outbreak of ranavirus in captive American bullfrogs (*Lithobates catesbeianus*) from Sinaloa, Mexico. The farm experienced high mortality in an undetermined number of juveniles and sub-adult bullfrogs. Affected animals displayed clinical signs and gross lesions such as lethargy, edema, skin ulcers, and hemorrhages consistent with ranavirus infection. The main microscopic lesions included mild renal tubular necrosis and moderate congestion in several organs. Immunohistochemical analyses revealed scant infected hepatocytes and renal tubular epithelial cells. Phylogenetic analysis of five partial ranavirus genes showed that the causative agent clustered within the Frog virus 3 clade. Risk assessment with the Pandora^+^ protocol demonstrated a high risk for the pathogen to affect amphibians from neighboring regions (overall Pandora risk score: 0.619). Given the risk of American bullfrogs escaping and spreading the disease to wild amphibians, efforts should focus on implementing effective containment strategies and surveillance programs for ranavirus at facilities undertaking intensive farming of amphibians.

## 1. Introduction

Mexico is the fifth ranking nation in terms of amphibian biodiversity with a total of 252 endemic species [1]. Moreover, the country ranks second in number of threatened amphibian species (*n* = 164) [1]. Main causes for amphibian population declines are anthropogenic activities such as deforestation and habitat fragmentation, and infectious diseases [2,3]. The two deadliest amphibian pathogens worldwide are the fungus *Batrachochytrium dendrobatidis* (Bd) and ranavirus [4]. The fungus Bd has caused epidemics in amphibian populations from mountainous regions of central and southern Mexico since the 1970s [5]. In contrast, ranaviruses (double-stranded DNA viruses from the family *Iridoviridae*) [4] have never been reported in Mexico, despite the wide distribution of these pathogens throughout the United States and Central and South America [6]. Insufficient surveillance is suspected to be the main reason for the lack of ranavirus records in Mexico [6].

Ranaviruses have been associated with die-offs of wild amphibians in America, where *Ambystoma tigrinum* virus (ATV) and frog virus 3 (FV3) are most prevalent [7,8]. In Europe, the common midwife toad viruses (CMTV) prevail in the wild [9,10,11], whereas in Asia, the tiger frog virus (TFV) and FV3 virus are present in the wild [12,13] and the CMTV strains have only been found in captive populations [14]. In many of these cases, ranavirus outbreaks have affected zoo collections [15], fisheries [16], or laboratory facilities [17].

The present study describes an outbreak of ranavirus disease in a captive colony of American bullfrogs (*Lithobates catesbeianus*) in the Mexican province of Sinaloa. Sinaloa is located in a transitional zone in the Pacific coast of Mexico and is home to at least 40 amphibian species, among them, salamanders of the families Ambystomatidae (1 species) and Plethodontidae (1 species), and anurans belonging to Bufonidae (9 species), Craugastoridae (4 species), Eleutherodactylidae (4 species), Hylidae (11 species), Leptodactylidae (1 species), Mycrohylidae (3 species), Pelobatidae (1 species), and Ranidae (5 species) [18]. Around 10% of the species from these families show evidence of population declines, 80% are considered stable and for the remaining 10% no information is available [1].

Commercial farming of American bullfrogs *(Lithobates catesbeianus*) started in Mexico during the 1950s when the species was introduced from the United States [19,20]. This species was first noticed in the wild in the northwestern part of Mexico in 1969 and has since become invasive and a major threat for ecological niches of endemic Mexican amphibians [19,21].

This work describes, for the first time, a ranavirus outbreak in farmed American bullfrogs (*Lithobates catesbeianus*) from Mexico. In late March of 2017, the rana-culture facility experienced a die-off involving an undetermined number of dead animals at various life stages, particularly juveniles and sub-adults. Macroscopic and microscopic lesions of affected animals, along with immunohistochemical and molecular analyses of the samples, revealed that the die-off was associated with an FV3-like ranavirus.

The aims of this study were to investigate the first ranavirus disease outbreak in Mexico and to analyze the potential risk that this pathogen poses for wild amphibian populations in neighboring areas.

## 2. Materials and Methods

### 2.1. General Information on the Rana-Culture Facility

**Figure 1:**
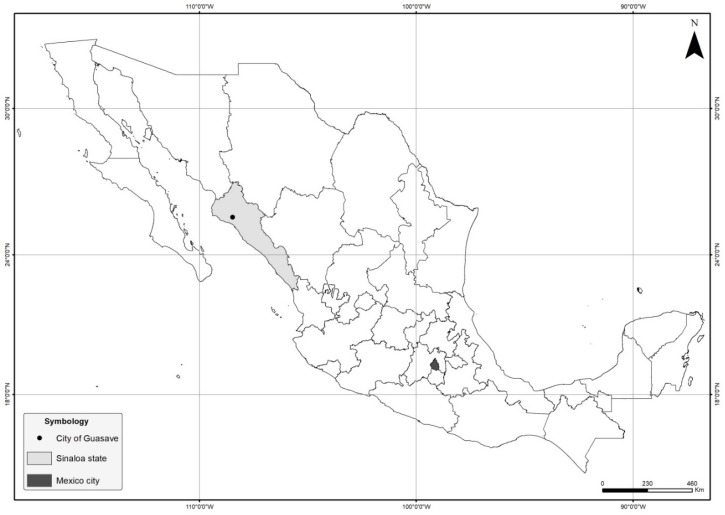
Location of city of the outbreak in Mexico. The city of Guasave is indicated by a black circle within the province of Sinaloa (grey). Mexico City, the place from which the bullfrogs were imported from, is shown in black.

The farm is located in the city of Guasave, province of Sinaloa (Figure 1). It started operations around 2009. The bullfrogs were originally brought in from a facility in central Mexico (state of Mexico) and were subsequently bred. Afterwards, a batch of frogs raised in the farm was bred to produce their own eggs and tadpoles, closing the culture system. The culture cycle normally lasts four to six months from tadpole to harvest size (120–250 g). The animals are used mainly for educational purposes and not for human consumption.

### 2.2. Necropsies and Sample Collection

Animals that reached humane end-points manifested as severe lesions and clinical signs (ulcerations and skin erythema, lethargy,) were euthanized with an overdose of barbiturates (by a licensed veterinarian). Necropsies of five bullfrogs were performed in situ and spleen, kidney, and liver were sampled for histopathology and PCR.

### 2.3. Histopathology and Immunohistochemistry

Fragments of spleen, kidney, and liver were stored in 10% formalin for 24 h. Subsequently, samples were dehydrated with ascending grades of ethyl alcohol (ETOH), embedded in paraffin blocks (Paraplast, Tissue Embedding Medium, MacCormick Scientific, Chicago, IL, USA), cut at 3 µm thickness and stained with hematoxylin and eosin (H&E) for microscopic examination [22]. Immunohistochemistry was performed based on an established protocol with a polyclonal rabbit anti-European catfish virus antibody (kindly donated by Dr. Anna Toffan, Instituto Zooprofilattico Sperimentale delle Venezie, Italy) with slight modifications [23]. Sections were dewaxed and hydrated through decreasing concentrations of alcohol, and gently washed twice with PBS, pH 7.2, for 5 min. Endogenous peroxidase activity was inhibited by immersion of the slides in 4% hydrogen peroxide for 30 min. Blocking was done by placing the slides on PBS buffer with 5% bovine serum albumin (BSA) for 30 min at room temperature. Antigen retrieval was achieved by incubating the slides with trypsin (0.1% working solution) at 37 °C for 20 min. Afterwards, slides were incubated with the polyclonal rabbit anti-European catfish virus antibody diluted to 1:1800 in PBS buffer with 2.5% BSA for 1 h at room temperature. After washing three times with PBS-Tween 0.1%, a biotinylated goat anti-rabbit IgG antibody (Vector, Burlingame, CA, USA) diluted to 1:250 in TBS buffer with 2.5% BSA was added and followed by a 30-minute incubation period. The slides were then washed with PBS-Tween three times, incubated with Avidin-Biotin Complex (Dako, Palo Alto, CA, USA) diluted to 1:250 in PBS buffer for 30 min at room temperature, washed with PBS for 5 min and a chromogen substrate (AEC) was added (Dako, Palo Alto, CA, USA) for 8 min. Afterwards, the slides were placed in running tap water for 5 min and counterstained with hematoxylin for 1 min. Finally, after a 10 min bath in running tap water, the slides were mounted with Aquatex (Merck, Kenilworth, NJ, USA) and examined under a light microscope. Negative controls included tissues from non ranavirus-infected amphibians, tissues omitting primary or secondary antibody and an isotype control.

### 2.4. DNA Extraction, PCR and Sequencing

DNA extraction was performed at the Laboratory of Pathology and Molecular Diagnosis (IPN) in Sinaloa, Mexico. This was done using 3% acetyl trimethyl ammonium bromide (CTAB) buffer (Tris HCl 100 mM pH 8.0, EDTA 20 mM, NaCl 1.4 M CTAB 3% *w*/*v*) added with 0.2% (*v*/*v*) β-mercaptoethanol [24].

Each sample was separately ground with a pestle in a 1.5 mL microtube and incubated at 60 °C for 30 min. Then, a mix (24:1) of chloroform-isoamyl alcohol was added to denature proteins and centrifuged (13,000 rpm, 10 min) to separate the aqueous phase containing nucleic acids. This was transferred to a new tube and nucleic acids were precipitated with cold isopropyl alcohol and centrifuged as above. The pellet was washed with cold 70% ethanol, centrifuged and supernatant decanted. Nucleic acids were air dried and dissolved in 70 µL nuclease-free water. Concentration was measured by nano-spectrophotometry (nanodrop 2000c, Thermo Scientific, Waltham, MA, USA).

For the PCR analyses, the primers used were those designed by Mao et al., which target a portion of the viral major capsid protein gene [25]. The PCR volume was 50 µL. A PCR mix was done using a DreamTaq DNA polymerase (Thermo Fisher Scientific, Waltham, MA, USA) containing 5 µL of DreamTaq PCR Green buffer (10×), the respective forward (1 µL) and reverse (1 µL) primer pairs and 40.5 µL nuclease-free water. One µL of DNA template was added to each reaction. A positive and a negative control (distilled water) were included.

Amplification conditions were: an initial DNA denaturation step at 95 °C for 3 min, followed by 35 cycles at 95 °C for 30 s, annealing at 49 °C for all primers used and extension at 72 °C for 60 s. A final extension at 72 °C for min was included. PCR products were resolved in a 1% agarose gel in tris HCl, acetic acid EDTA (TAE) buffer and visualized in a Gel Doc XR+ documentation system (BioRad, Inc., Hercules, CA, USA).

The resulting PCR products were purified using a ExoSAP-IT^®^ PCR Product Cleanup kit (GE Healthcare, Santa Clara, CA, USA) and sent for Sanger sequencing at (Macrogren, Amsterdam, NL, USA). The sequence was used for multiple sequence alignment and phylogenetic analyses.

### 2.5. Phylogenetic Analysis

For phylogenetic analysis, five partial ranavirus genes were selected (13R, 16L/MCP, 22L, 59R and 82L). The partial gene sequences of the Mexican ranavirus were compared to those of 20 other members of the family *Iridoviridae*. The DNA sequences were aligned using the software CLUSTAL Omega with default settings (https://www.ebi.ac.uk/Tools/msa/clustalo/) [26]. Maximum likelihood phylogeny was re-constructed with the software MEGA 6.0 using 1000 bootstrap replicates. The best-fit model Kimura 2 parameter + Gamma distribution (K2 + G) was chosen based on the lowest Bayesian Information Criterion score.

### 2.6. Risk Classification Using the PANDORA + Protocol

To assess the risks that ranavirus would pose for the wild endemic amphibians in Sinaloa, Mexico, the Pandora^+^ protocol was used [27]. This protocol is specific for invasive microbes and complies with EU regulations for risk assessment that supports the listing of invasive alien species. This program originates from the Invasive Species Environmental Impact Assessment protocol (ISEIA) [28]. The ISEIA protocol was previously used to perform a risk analysis on ranaviruses in The Netherlands [29]. The protocol consists of a questionnaire (20 questions) covering distinct modules which include entry, exposure, and consequences for various targets including environment, plants, domestic animals, and humans. Each question classifies the risk as low, medium, or high with a corresponding low, medium, or high level of confidence. Default settings of the Pandora^+^ program were used. In the default settings, each alternative answer to a question from a module is ranked 0–1 (lowest to highest) and then the arithmetic mean of the questions are calculated to obtain a value for each module. An equal weight value of 1 was allocated to each of the questions. The exposure/entry score is calculated by taking the geometric mean of the emerging/entry and emerging/exposure modules. The scores from the different impact/consequence modules are similarly aggregated to calculate a general consequence score by using the geometric mean from each module. Finally, the overall risk score is calculated by multiplying the exposure/entry score by the consequence score. The overall risk score may be: low (0–0.25), medium (0.25–0.50), high (0.50–0.75), and very high (0.75–1.00). Four of the coauthors, each with a particular area of expertise (biology, virology, veterinary pathology, and ecology) performed the risk assessment separately and later unified criteria to provide a consensus risk score.

### 2.7. Level of Wild Species Threat

The level of ranavirus infection threat from each of the amphibian species within the province of Sinaloa was evaluated by consulting the species status in the IUCN Red List (International Union for Conservation of Nature) [1] and the Environmental Vulnerability Score (EVS), which is an algorithm comprised of three scales of added values which consist of geographical distribution (score range 1–6), ecological distribution based on types of forest formations (score range 1–8), and reproductive mode which takes into account type of wetlands where larvae develop and in which egg masses are laid (score range 1–5). Once these elements are added, the score range can be from 3 to 19, this allows to categorize the threat as low (3–9), medium (10–13), and high (14–19) [30].

## 3. Results

**Figure 2:**
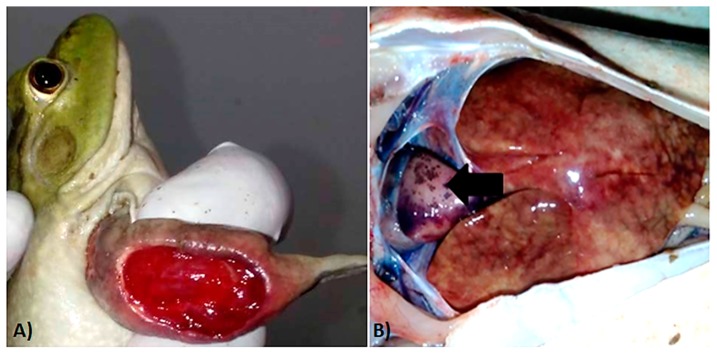
Macroscopic lesions of ranavirus-infected bullfrogs. (**A**) Extensive ulceration of the forelimb. (**B**) Extensive hepatic necrosis and epicardial pallor and hemorrhages (black arrow).

External macroscopic lesions of affected individuals included skin hemorrhages and ulcerations (Figure 2A), while internal inspection of the coelomic cavity revealed multiple lesions including hepato-splenomegaly, hepatic necrosis, and epicardial hemorrhages (Figure 2B).

**Figure 3:**
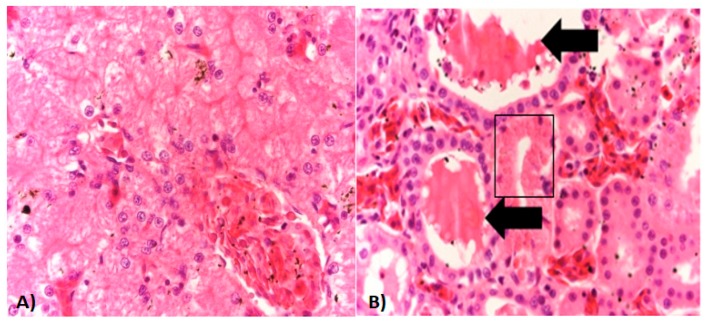
Histopathology of ranavirus-infected American bullfrogs. (**A**) Liver section from an affected bullfrog with mild congestion of sinusoids. (**B**) Kidney section shows an area of mild tubular necrosis characterized by cytoplasmic hyper-eosinophilia, loss of nuclei, and mild nuclear pyknosis (black square). Intraluminal protein casts are also seen (black arrows). Hematoxylin/Eosin staining. Magnification for all photos 20×/100 μm.

Most of the microscopic lesions in organs from examined specimens were moderate and consisted primarily of congestive changes (Figure 3A) with the exception of the kidney which had multifocal areas of mild tubular necrosis and intraluminal eosinophilic protein casts (Figure 3B).

**Figure 4:**
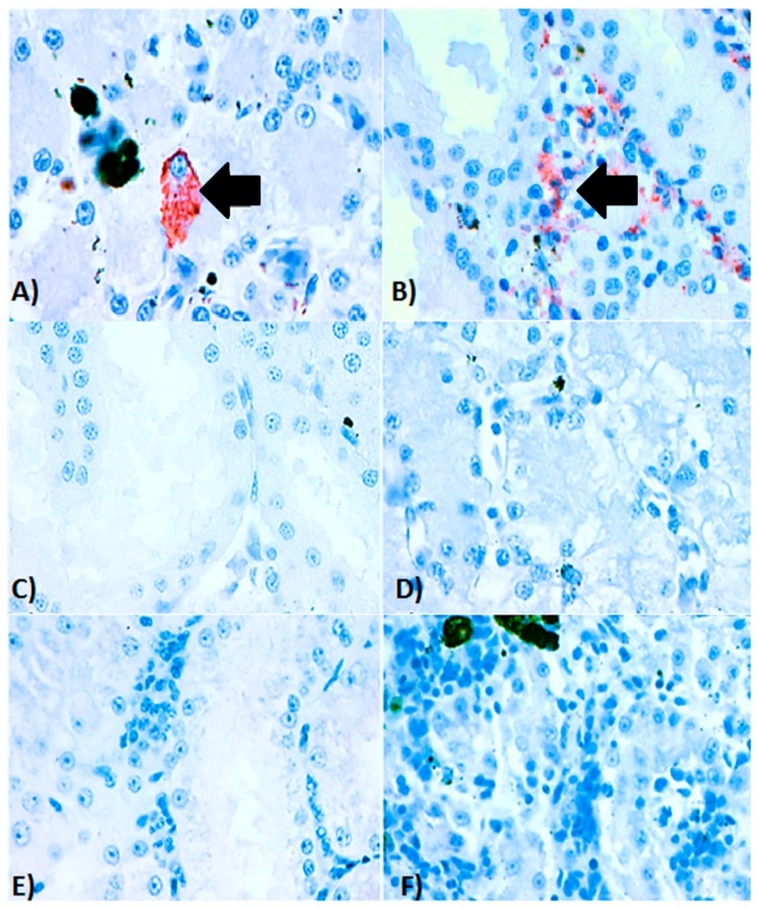
Immunohistochemistry of ranavirus-infected American bullfrogs (*Lithobates catesbeianus*). (**A**) Immunohistochemical staining of kidney, in which positive immunolabelling (red staining) is present in the renal cortical interstitium along with areas of necrosis and scant inflammation. (**B**) Immunohistochemical staining of a liver from an affected bullfrog with a focal area of positive immunolabelling (red staining) in the cytoplasm of a Kupffer cell. (**C**) Serial section of the kidney from the same animal without secondary antibody (control), (**D**) Serial section of liver from the same animal without secondary antibody (control). (**E**) Histological kidney section from an anuran which was PCR negative for ranavirus infection (control). (**F**) Histological liver section from an anuran which was PCR negative for ranavirus infection (control). Magnification for all photos 40×/50 μm.

**Figure 5:**
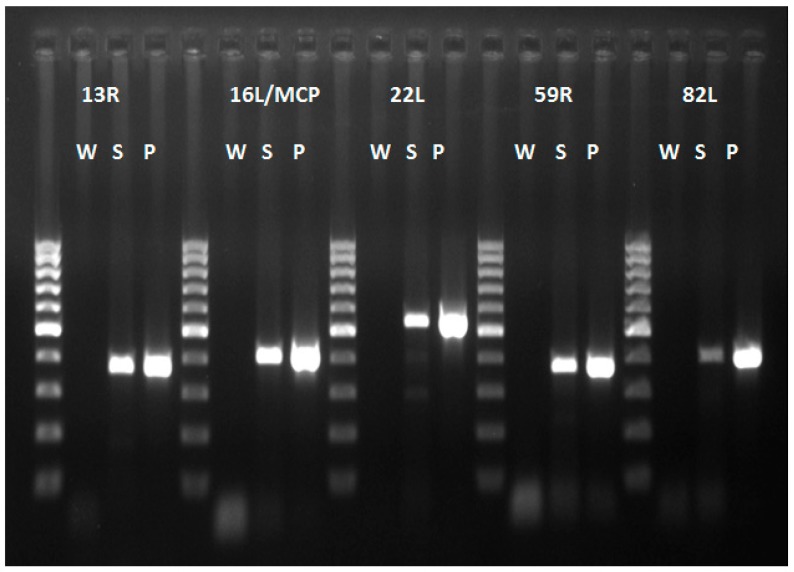
PCR products of five ranavirus genes. All products of the Mexican virus samples (S) are of similar size to those of the FV3 virus positive control (P). Abbreviations: W (water), S (Mexican virus sample), P (Positive control frog virus 3 isolate *Oophaga pumilio*/2015/Netherlands/UU3150324001 (GenBank no. MF360246)). 100 bp ladder.

**Figure 6:**
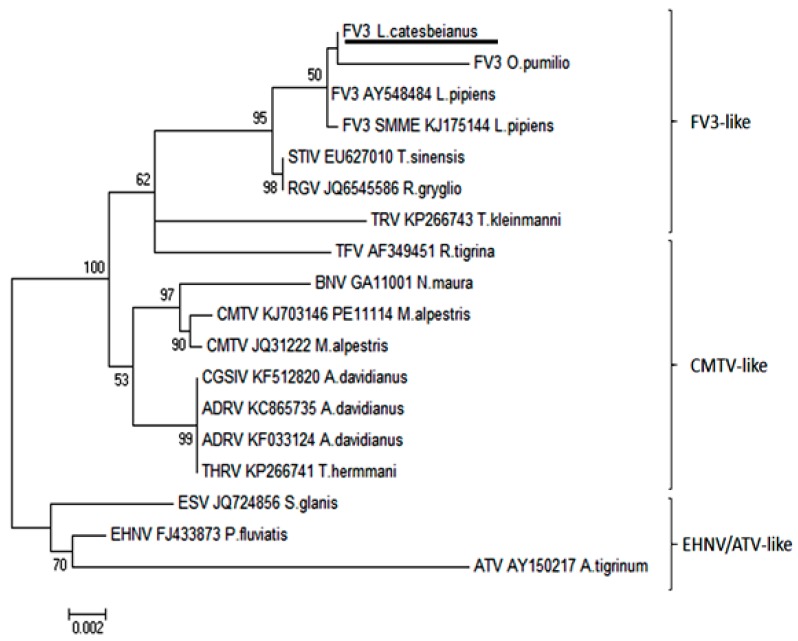
Phylogeny of five partial ranavirus gene sequences (1000 bootstrap values). The Mexican isolate FV3 *L. catesbeianus* (underlined) clusters closely within the FV3 ranavirus clade. Isolates and Genbank numbers used: Frog virus 3/*Lithobates catesbeianus*/2018/Mexico (GenBank accession numbers for partial sequences available in Supplementary Table S1), frog virus 3 (AY548484), frog virus 3 isolate SMME (KJ175144), tortoise ranavirus isolate 1 (882/96) (KP266743), Bosca’s newt virus isolate GA11001 (KJ703118), frog virus 3 isolate *Oophaga pumilio*/2015/Netherlands/UU3150324001 (MF360246), common midwife toad ranavirus isolate *Mesotriton alpestris*/2008/E (JQ231222), common midwife toad virus isolate P11114 (KJ703146), *Testudo hermanni* ranavirus isolate CH8/96 (KP266741), tiger frog virus (AF389451), soft-shelled turtle iridovirus (EU627010), European sheatfish virus (JQ724856), epizootic hematopoietic necrosis virus (FJ433873), *Ambystoma tigrinum stebbensi* virus (AY150217), *Andrias davidianus* ranavirus isolate (KF033124), *Andrias davidianus* ranavirus isolate 1201 (KC865735), Chinese giant salamander iridovirus, isolate CGSIV-HN1104, (KF512820), and *Rana grylio* virus (JQ654586). Only bootstrap values higher than 50 are shown.

Intracytoplasmic inclusion bodies, which are considered the microscopic hallmark of ranavirus disease [9,10], were not observed in any of the specimens examined. Areas of positive immunohistochemical staining were scant and were only found in a few Kupffer cells of the liver (Figure 4A) and in the cortical interstitium of the kidney (Figure 4B). Initial PCR on samples from five frogs showed mild to strong positive signal in both liver and kidney of three frogs, mild positive signal in the liver of one frog, and one frog negative in all organs PCR bands for all five partial genes were from similar size to an FV3 isolate used as a positive control (GenBank no. KT003504) [31] (Figure 5). Phylogeny of five partial ranavirus genes revealed that the Mexican isolate clustered within the FV3 group and was closely related to USA isolates FV3 (AY548484) [32], FV3 SSME (KJ175144) [33], and a Nicaraguan isolate (MF360246) [31] (Figure 6). Sanger sequencing of a portion of the major capsid protein (462 bp) [25] showed that the ranavirus involved in this outbreak shared a 100% nucleotide homology with the original frog virus 3 isolate from the United States (AY 548484) [31]. The GenBank accession numbers for the Mexican virus sequences and the genomic identity to the ranavirus strains used for phylogeny are available as Supplementary Table S1.

The results of the Pandora^+^ protocol showed a high overall risk score for endemic amphibians in neighboring areas (overall risk score 0.619, confidence level 1). The risk of entry of the pathogen through animals harboring subclinical infections or through virions in water was found to be medium (risk score 0.5, confidence level 1), given that waste water disposal of the farm is through the general sewage system without the water undergoing any prior disinfection treatment. Additionally, there is also the possibility of human-mediated transport and subsequent introduction of the virus if virions persist in footwear from employees. The possibilities of the pathogen to emerge, become established, and affect native amphibian populations were also found to be high (consequence score 0.875, confidence level 1) due to susceptible amphibian species present in the area. Since the pathogen does not affect plants or warm-blooded domestic animals or humans, the risk score for all these factors was classified as inapplicable.

Finally, given the potential effects of ranavirus on the ecosystem, like the disappearance of endangered amphibian specimens or the possible increase in insect plagues, there is a low chance that the pathogen may have an indirect effect on tourism of the area. Furthermore, even though there is no current indication that the farm exports frogs out of Mexico, the risk of virus introduction through trade of sub-clinically infected animals cannot be entirely discarded (low risk, score 0.25, confidence score 0.5). A summary of the results of the scoring and confidence values is provided in Table 1. The original questionnaires and final risk scores provided by all four experts are available as Supplementary Tables S2–S5.

Comparison of the information on the threat categories for all 40 amphibian species in Sinaloa based on the IUCN list and the environmental vulnerability scores (EVS) revealed moderate differences in terms of threat classification. The IUCN list showed that 77.5% of the species were categorized as of least concern, 10% of them were not listed, 5% were considered vulnerable, 5% were classified as endangered and 2.5% as nearly threatened. On the other hand, in terms of environmental vulnerability, the EVS system categorized 45% of the species as having low vulnerability, 40% as medium, and 20% as high. All families to which the species belonged to, except for Eleutherodactylae and Microhylidae, have been reported to be affected by ranaviruses [6]. Out of the 19 amphibian species within 50 km or less of the original outbreak, the Tarahumara frog (*Lithobates tarahumarae*) and the Bell’s salamander (*Isthmura bellii*) were considered vulnerable and the others as least concern by the IUCN red list. With the EVS system, the little Mexican toad (*Anaxyrus kelloggi*) was considered at high risk (>14 EVS), eight other species were considered as medium risk and the rest as low risk. Information regarding the level of threat of amphibian species in Sinaloa and the approximate distance of species distribution to the outbreak site can be found in Table 2.

## 4. Discussion

This study investigated the first outbreak of ranavirus infection in Mexico and performed a risk analysis for the endemic wild amphibian species in the area close to the affected farm. The ranavirus outbreak took place in a farmed colony of American bullfrogs and was caused by a ranavirus clustering within the FV3 group, the dominant clade of ranaviruses present on the American continent [6].

One of the most concerning aspects of American bullfrogs is their potential to introduce high-risk pathogens such as ranavirus and *Batrachochytrium dendrobatidis* into amphibian populations [34]. Breeding facilities for these animals are considered hotspots for ranavirus outbreaks as shown in various countries such as Brazil and Japan [35,36]. Frequently, ranavirus is introduced to captive amphibian populations by means of capture of infected animals from the wild. This has taken place in Switzerland, in which a ranavirus-outbreak in a laboratory colony of water frogs (*Pelophylax* spp.) was shown to originate from a ranavirus-infected frog captured from a natural pond in Germany [17].

Despite the severe gross lesions (skin ulceration and erythema), affected animals sampled from this farm showed only scant immunohistochemical signal in the liver and kidney and no intracytoplasmic inclusion bodies in histology. The lack of inclusion bodies is a well-known feature of bullfrog ranavirus-associated outbreaks in farms [35,36]. The faint immunohistochemical signal observed in the kidneys and liver could be consistent with a chronic presentation of the disease where the most severe lesions have probably resolved in visceral organs, but skin lesions persist as severe ulcerations. This chronic presentation of the disease has also been known as “ulcerative syndrome”, reported in common frogs (*Rana temporaria*) in the United Kingdom infected with FV3-like ranavirus isolates [37]. Investigation focusing on immunohistochemical analyses on animals with this condition revealed overall decrease in signal on visceral organs in comparison to animals with the per-acute or acute presentations of the disease, known as “hemorrhagic syndrome” [37].

Independent assessment of introduction and establishment risk of ranaviruses into Mexican wildlife by all four experts showed similar scoring results in the Pandora + protocol. Consensus analysis suggested that the possibilities of the pathogen being introduced into nature by bullfrogs escaping the facility was moderate (0.5). Bullfrogs are quick to colonize new habitats and capable of long-distance dispersal (>1200 meters in a week) [38]. This phenomenon has occurred precisely in the study area.

In 1956, the American bullfrog was introduced for exploitation as a harvestable food item in the vicinity of Los Mochis, Ahome (approximately 60 km north of Guasave) [39]. Only six years later, there were several populations among Ahome and Guasave counties established on agricultural irrigation channels and in ponds [40]. Such a colonization presents a threat to native amphibian species as it enables bullfrogs to become into direct contact with susceptible hosts.

Other than introduction through infected hosts, ranavirus can also gain entry into the wild through virion-contaminated water [41]. At the facility where the outbreak took place, untreated water is usually disposed through the general sewage system. It is possible that this potentially contaminated water could reach natural ponds and pools which are home to susceptible host species and are thus apt for ranavirus transmission and persistence.

The probability of the virus becoming endemic within native amphibian populations was found to be high mainly due to the amphibian species present in the area. In total, 17 species are found in the county of Guasave, and most of them live permanently or reproduce in the water, except for Barking frog (*Craugastor augusti*) that reproduces and lives in a terrestrial environment. Among these species, the little Mexican toad (*Anaxyrus kelloggi*) was found to possess a high vulnerability score [30]. Members of the family Bufonidae, to which the little Mexican toad belongs to, have been known to develop ranavirus-associated hemorrhagic syndrome when exposed to FV3 ranavirus strains in the UK [37].

The two families of anurans in which the highest amount of infected species have been reported are Ranidae and Hylidae [6]. Within the Ranidae family, one of the most prevalent amphibian species in the region is the Northwest Mexican leopard frog (*Lithobates magnaocularis*) and one of the most vulnerable ones according to the EVS is the Tarahumara frog [30].

Ranavirus has been able to establish itself among populations of other amphibian species from the genus *Lithobates*, like the Northern leopard frog (*Lithobates pipiens*) [42] and the Dusky gopher frog (*Lithobates sevosus*), in which it has caused 100% mortality under experimental settings [43]. Regarding the family Hylidae, and in contrast to all other endemic amphibian species in Guasave, the Mexican leaf frog (*A. dacnicolor*) has actually shown evidence of population declines throughout Mexico [1]. This is of particular concern, since other members of Hylidae, like the magnificent tree frog (*Litoria splendida*) and green tree frog (*Hyla cinerea*) have suffered severe outbreaks of ranavirus disease under captive settings [44]. Likewise, larval stages of the Cope’s gray tree frog (*Dryophytes chrysoscelis*) have shown a high mortality rate upon experimental exposure to FV3 ranavirus highlighting the species susceptibility to develop lethal infections [45].

However, the risk of ranavirus infection is not only limited to amphibians, and the possibility of transmission from infected amphibians to susceptible fish in neighboring ponds is supported by previous evidence of ranavirus outbreaks affecting both fish and frogs and experimental interspecies transmission of ranaviruses. In the wild in the USA, an FV3-like virus caused a die-off simultaneously involving threespine stickleback fish (*Gasterosteus aculeatus)* and red-legged frogs (*Rana aurora*) [46]. Under experimental conditions, Bohle iridovirus (BIV), a ranavirus originally isolated from the ornated burrowing frog (*Limnodynastes ornatus*), induced infection and mortality of barramundi fish (*Lates calcarifer*) [47].

There is also a high probability of the virus to persist in the area due to suitable environmental conditions. The climate of the region where the outbreak took place is mostly warm and dry with an average temperature of 24 °C which can surpass 30 °C in the summer months [48]. Warm temperatures of around 20 °C, have been shown to favor ranavirus propagation and worsen signs of ranavirus disease under experimental settings [49]. Additionally, virions of Bohle iridovirus, an FV3-like ranavirus, have been known to persist in desiccated soil under a temperature of 45 °C [50]. Research has also shown that virions are able to remain infectious in waterbodies for up to one or two months in water temperatures ranging from 4 °C to 20 °C [41].

There are several measures which could be undertaken to prevent further spread into the wild. Most importantly, culling and re-stocking of animals within the farm would ensure eradication of not only animals harboring active infections but also of sub-clinically infected carriers.

Subsequent disinfection of the facilities, equipment or footgear of employees and especially waste-water treatment with cleaning agents like Virkon (at a 1% concentration) would aid in eliminating infectious virions present in the water or soil. Monitoring of amphibian populations and molecular analysis of skin swabs from a subset of animals from nearby ponds has shown to be an effective way of gaining information regarding genotype, virulence, and probability of further spread [51]. Another efficient way of monitoring and eventually controlling potential ranavirus disease outbreaks is by informing and encouraging the public to report amphibian die-offs or to keep tract of the number and life-stages of these animals in nearby ponds. Long-term science projects involving citizens have proven to be a successful approach to studying and characterizing this disease in wild amphibians in Great Britain [52].

The current study characterized the first ranavirus outbreak in captive Mexican amphibians through pathology and molecular biology. It also determined a high level of risk of the pathogen to gain entry and become established in neighboring nature areas and potentially affect endemic amphibian species of conservation concern.

## Figures and Tables

**Table 1:** Consensus risk classification of frog virus 3 ranavirus in Mexico. The Pandora^+^ questionnaire was solved as a consensus after all four experts had provided their individual results and discussed answers. Total number of questions: 20. Out of these, six questions comprised the initial assessment, one endemic exposure, one emerging/entry, two emerging/exposure, and two questions per each module of impact (environment, plants, animal, human and other). Color scheme and risk classification is based on cut-off values for scores by Schiphouwer et al. [28].

Module	Risk Classification	Risk Score	Certainty	Confidence
Emerging/entry	Medium	0.5	Medium	0.5
Emerging/exposure	High	1	Medium	0.75
Environmental score	High	0.875	High	1
Plant score	n/a	0	High	1
Animal score	n/a	0	High	1
Human score	n/a	0	High	1
Other score	Low	0	Medium	0.5
Consequence	High	0.875		
Entry exposure	High	0.707		
Risk score	High	0.619		

n/a inapplicable.

**Table 2:** Level of threat for native amphibian species within the province of Sinaloa. The table summarizes the endemic, IUCN, and EVS, status and estimated distance of the nearest population of specific species to the affected farm.

Family	Scientific Name	Common Name	Endemic	IUCN Status	EVS Score	Distance to Farm
Ambystomatidae	*Ambystoma rosaceum*	Tarahumara salamander	Yes	LC	14	100–200 km
Bufonidae	*Anaxyrus cognatus*	Great plains toad	No	LC	9	100–200 km
Bufonidae	*Anaxyrus kelloggi*	Little mexican toad	Yes	LC	14	<50 km
Bufonidae	*Anaxyrus mexicanus*	Mexican spadefoot toad	Yes	NT	13	100–200 km
Bufonidae	*Anaxyrus punctatus*	Red-spotted toad	No	LC	5	<50 km
Bufonidae	*Incilius alvarius*	Sonoran desert toad	No	LC	11	<50 km
Bufonidae	*Incilius marmoreus*	Marbled toad	Yes	LC	11	>200 km
Bufonidae	*Incilius mazatlanensis*	Sinaloan toad	Yes	LC	12	<50 km
Bufonidae	*Incilius occidentalis*	Pine toad	Yes	LC	11	>200 km
Bufonidae	*Rhinella marina*	Cane toad	No	LC	3	<50 km
Craugastoridae	*Craugastor augusti*	Barking frog	No	LC	8	<50 km
Craugastoridae	*Craugastor horbartsmithii*	Smith’s pigmy robber frog	Yes	E	15	>200 km
Craugastoridae	*Craugastor occidentalis*	Taylor’s barking frog	Yes	NL	13	>200 km
Craugastoridae	*Craugastor vocalis*	Pacific stream frog	Yes	LC	13	<50 km
Eleutherodactylae	*Eleutherodactylus interorbitalis*	Spectacled chirping frog	Yes	NL	15	100–200 km
Eleutherodactylae	*Eleutherodactylus nitidus*	Shiny peeping frog	Yes	LC	12	>200 km
Eleutherodactylae	*Eleutherodactylus saxatilis*	Marbled peeping frog	Yes	E	17	>200 km
Eleutherodactylae	*Eleutherodactylus teretistes*	Whistling frog	Yes	NL	16	>200 km
Hylidae	*Agalychnis dacnicolor*	Mexican leaf frog	Yes	LC	13	<50 km
Hylidae	*Dryophytes arenicolor*	Canyon treefrog	No	LC	7	>100 km
Hylidae	*Dryophytes eximius*	Mountain treefrog	Yes	LC	10	>200 km
Hylidae	*Dryophytes wrightorum*	Wright’s mountain treefrog	No	LC	9	>200 km
Hylidae	*Exerodonta smaradigna*	Emerald treefrog	Yes	LC	12	>200 km
Hylidae	*Sarcohyla bistincta*	Mexican Fringed-limbed treefrog	Yes	LC	9	>200 km
Hylidae	*Smilisca baudinii*	Common mexican treefrog	No	LC	3	<50 km
Hylidae	*Smilisca fodiens*	Lowland burrowing treefrog	No	LC	8	<50 km
Hylidae	*Tlalocohyla smithii*	Dwarf mexican treefrog	No	LC	11	<50 km
Hylidae	*Trachycephalus typhonius*	Veined treefrog	No	LC	4	>200 km
Hylidae	*Triprion spatulatus*	Mexican shovel-headed treefrog	Yes	LC	13	>200 km
Leptodactylae	*Leptodactylus melanotonus*	Black-backed frog	No	LC	6	<50 km
Mycrohylidae	*Gastrophryne mazatlanensis*	Mazatlan narrowmouth frog	No	NL	8	<50 km
Mycrohylidae	*Hypopachus ustus*	Two-spaded narrow-mouthed toad	No	LC	7	100–200 km
Mycrohylidae	*Hypopachus variolosus*	Mexican narrow-mouthed toad	No	LC	4	100–200 km
Pelobatidae	*Scaphiopus couchii*	Couch’s spadefoot toad	No	LC	3	<50 km
Plethodontidae	*Isthmura bellii*	Bell’s salamander	Yes	V	12	50–100 km
Ranidae	*Lithobates catesbeianus*	American bullfrog	No	LC	10	<50 km
Ranidae	*Lithobates forreri*	Forrer’s leopard frog	No	LC	3	<50 km
Ranidae	*Lithobates magnaocularis*	Northwest mexican leopard frog	Yes	LC	12	<50 km
Ranidae	*Lithobates pustulosus*	Mexican cascades frog	Yes	LC	9	>200 km
Ranidae	*Lithobates tarahumarae*	Tarahumara frog	Yes	V	8	50–100 km

LC least concern, E, endangered, NL, not listed, NT, near threatened, V, vulnerable.

## Supplementary Materials

The following are available online at http://www.mdpi.com/1999-4915/11/1/26/s1, Table S1: Comparison of genomic identity between Mexican ranavirus and other ranaviruses, Tables S2–S5: Risk assessment 2–5.

## References

[1] IUCN 2017 The IUCN Red List of Threatened Species. http://www.iucnredlist.org.

[2] Bower D.S., Lips K.R., Schwarzkopf L., Georges A., Clulow S. (2017). Amphibians on the brink. Science.

[3] Blaustein A.R., Kiesecker J.M. (2002). Complexity in conservation: Lessons from the global decline of amphibian populations. Ecol. Lett..

[4] Chinchar V.G. (2002). Ranaviruses (family *Iridoviridae*): Emerging cold-blooded killers. Arch. Virol..

[5] Mendoza-Almeralla C., Burrowes P., Parra-Olea G. (2015). Chytridiomycosis in amphibians from Mexico: A revision. Rev. Mex. Biodivers..

[6] Duffus A.L.J., Waltzek T.B., Gray M.J., Chinchar G.V. (2015). Distribution and Host Range of Ranaviruses. Ranaviruses: Lethal Pathogenics of Ectothermic Vertebrates.

[7] Epstein B., Storfer A. (2016). Comparative genomics of an emerging amphibian virus. G3 Genes Genom. Genet..

[8] Wheelwright N.T., Gray M.J., Hill R.D., Miller D.L. (2014). Sudden mass die-off of large population of wood frog (*Lithobates sylvaticus*) tadpoles in Maine, USA, likely due to ranavirus. Herpetol. Rev..

[9] Balseiro A., Dalton K., del Cerro A., Marquez I., Cunningham A.A., Parra F., Prieto J.M., Casais R. (2009). Pathology, isolation and molecular characterisation of a ranavirus from the common midwife toad *Alytes obstetricans* on the Iberian Peninsula. Dis. Aquat. Org..

[10] Kik M., Martel A., van der Sluijs A.S., Pasmans F., Wohlsein P., Gröne A., Rijks J.M. (2011). Ranavirus-associated mass mortality in wild amphibians, The Netherlands, 2010: A first report. Vet. J..

[11] Miaud C., Pozet F., Gaudin N.C.G., Martel A., Pasmans F., Labrut S. (2016). Ranavirus causes mass die-offs of alpine amphibians in the Southwestern Alps, France. J. Wildl. Dis..

[12] He J.G., Lu L., Deng M., He H.H., Weng S.P., Wang X.H., Zhou S.Y., Long Q.X., Wang X.Z., Chan S.M. (2002). Sequence analysis of the complete genome of an iridovirus isolated from tiger frog. Virology.

[13] Lei X.Y., Ou T., Zhu R.L., Zhang Q.J. (2012). Sequencing and analysis of the complete genome of *Rana grylio* virus (RGV). Arch. Virol..

[14] Geng Y., Wang K.Y., Zhou Z.Y., Li C.W., Wang J., He M., Yin Z.Q., Lai W.M. (2011). First report of a ranavirus-associated with morbidity and mortality in farmed Chinese giant salamanders (*Andrias davidianus*). J. Comp. Pathol..

[15] Marschang R.E., Becher P., Posthaus H., Wild P., Thiel H.J., Müller-Doblies U., Kalet E.F., Bacciarini L.N. (1999). Isolation and characterization of an iridovirus isolated from Hermann’s tortoises (*Testudo hermanni*). Arch. Virol..

[16] Whittington R.J., Kearns C., Hyatt A.D., Hengstberger S., Rutzou T. (1996). Spread of *Epizootic hematopoietic necrosis virus* (EHNV) in red fin perch (*Perca fluviatilis*) in southern Australia. Aust. Vet. J..

[17] Stöhr A.C., Hoffmann A., Papp T., Robert N., Pruvost N.B.M., Reyer H.U., Marschang R.E. (2013). Long-term study of an infection with ranaviruses in a group of edible frogs (*Pelophylax* kl. *esculentus*) and partial characterization of two viruses based on four genomic regions. Vet. J..

[18] Serrano J.M., Berlanga-Robles C.A., Ruiz-Luna A. (2014). High amphibian diversity related to unexpected environmental values in a biogeographic transitional area in north-western Mexico. Contrib. Zool..

[19] Casas-Andreu G., Aguilar-Miguel X., Cruz-Aviña R. (2001). La introducción y el cultivo de la rana toro (*Rana catesbeiana*). ¿Un atentado a la biodiversidad de México?. Revista Científica Multidisciplinaria de Prospectiva.

[20] Cifuentes-Lemus J.L., Torres-García M.P., Frías-Mondragón M. (1997). El Océano y Sus Recursos XI. Acuicultura.

[21] Luja V.H., Rodrìguez-Estrella R. (2010). The invasive bullfrog *Lithobates catesbeianus* in oases of Baja California Sur Mexico: Potential effect in a fragile ecosystem. Biol. Invasions.

[22] Morales-Salinas E., Aguilar-Arriaga B.O., Ramírez-Lezama J., Méndez-Bernal A., López-Garrido S. (2013). Oral fibrosarcoma in a black iguana (*Ctenosaura pectinata*). J. Zoo Wildl. Med..

[23] Rijks J.M., Saucedo B., Spitzen-van der Sluijs A.M., Wilkie G.S., van Asten A.J.A.M., van den Broek J., Boonyarittichaikij R., Stege M., Van der Sterren F., Martel A. (2016). Investigation of amphibian mortality events in wildlife reveals an on-going ranavirus epidemic in the North of The Netherlands. PLoS ONE.

[24] Zhang Y., Uyemoto J.K., Kirkpatrick B.C. (1998). A small-scale procedure for extracting nucleic acids from woody plants infected with various phytopathogens for PCR assay. J. Virol. Methods.

[25] Mao J., Hendrick R.P., Chinchar V.G. (1997). Molecular characterization, sequence analysis, and taxonomic position of newly isolated fish iridoviruses. Virology.

[26] CLUSTAL Omega. https://www.ebi.ac.uk/Tools/msa/clustalo/.

[27] D’hondt B., Vanderhoeven S., Roelandt S., Mayer F., Versteirt V., Ducheyne E., San Martin G., Grégoire J.C., Stiers I., Quoilin S. (2014). Pandora: A Risk Screening Tool for Pathogens and Parasites.

[28] Schiphouwer M.E., Felix R.P.W.H., van Duinen G.A., de Hoop L., de Hullu P.C., Matthews J., van der Velde G., Leuven R.S.E.W. (2017). Risk assessment of the alien smallmouth bass (*Micropterus dolomieu*). Rep. Environ. Sci..

[29] Rijks J.M., Spitzen van-der Sluijs A., Leuven R.S.E.W., Martel A., Kik M., Pasmans F. (2012). Risk Analysis of the Common Midwife Toad-Like Virus (CMTV-Like Virus) in The Netherlands.

[30] Wilson L.D., Johnson J.D., Mata-Silva V. (2013). A conservation reassessment of the amphibians of Mexico based on the EVS measure. Amphib. Reptile Conserv..

[31] Saucedo B., Hughes J., Súarez N., Haenen O., Kik M.J.L., van Beurden S.J. (2017). Complete genome sequence of frog virus 3 isolated from a Strawberry poison frog (*Oophaga pumilio*) imported from Nicaragua into The Netherlands. Genome Announc..

[32] Tan W.G., Barkman T.J., Chinchar G.V., Essani K. (2004). Comparative genomic analyses of frog virus 3, type species of the genus *Ranavirus* (family *Iridoviridae*). Virology.

[33] Morrison E.A., Garner S., Echaubard P., Lesbarreres D., Kyle C.J., Brunetti C.R. (2014). Complete genome analysis of a frog virus 3 (FV3) isolate and sequence comparison with isolates of differing levels of virulence. Virol. J..

[34] Kolby J.E., Smith K.M., Berger L., Karesh W.B., Preston A., Pessier A.P., Skerratt L.F. (2014). First evidence of amphibian chytrid fungus (*Batrachochytrium dendrobatidis*) and ranavirus in Hong Kong amphibian trade. PLoS ONE.

[35] Mazzoni R., Mesquita A.J., Fleury L.F.F., Brito W., Nunes I.A., Robert J., Morales H., Coelho A.S.G., Barthasson D.L., Galli L. (2009). Mass mortality associated with a Frog virus 3-like ranavirus infection in farmed tadpoles *Rana catesbeiana* from Brazil. Dis. Aquat. Org..

[36] Une Y., Sakuma A., Matsueda H., Nakai K., Murakami M. (2008). Ranavirus outbreak in North American bullfrogs (*Rana catesbeiana*), Japan, 2008. Emerg. Infect. Dis..

[37] Cunningham A.A., Temns C.A., Russel P.H. (2008). Immunohistochemical demonstration of ranavirus antigen in the tissues of infected frogs (*Rana temporaria*) with systemic hameorrhagic or cutaneous ulcerative disease. J. Comp. Pathol..

[38] Willis Y.L., Moyle P.B., Basket T.S. (1956). Emergence, breeding, hibernation, movements and transformation of the bullfrog, *Rana catesbeiana*, in Missouri. Copeia.

[39] Aguilar Ibarra F. (1963). Aspectos Generales sobre las Ranas y su Cultivo. Trab. Div. Inst. Nac. Invest. Biol. Pesq. C.

[40] Hardy L.M., McDiarmid R.W. (1969). The amphibians and reptiles of Sinaloa, Mexico. Univ. Kansas Publ. Mus. Nat. Hist..

[41] Nazir J., Spengler M., Marschang R.E. (2012). Environmental persistence of amphibian and reptilian ranaviruses. Dis. Aquat. Org..

[42] Echaubard P., Paulli B.D., Trudeau V.L., Lesbarrères D. (2015). Ranavirus infection in northern leopard frogs: The timing and number of exposures matter. J. Zool..

[43] Earl J.E., Chaney J.C., Sutton W.B., Lillard C.E., Kouba A.J., Langhorne C., Krebs J., Wilkis R.P., Hill R.D., Miller D.L. (2016). Ranavirus could facilitate local extinction of rare amphibian species. Oecologia.

[44] Jerett I.V., Whittington R.J., Weir R.P. (2015). Pathology of a Bohle-like infection in two Australian frog species *(Litoria splendida* and *Litoria caerulea*). J. Comp. Pathol..

[45] Brand M.D., Hill R.D., Brenes R., Chaney J.C., Wilkes R.P., Grayfer L., Miller D.L., Gray M.J. (2016). Water temperature affects susceptibility to ranavirus. Ecohealth.

[46] Mao J.D.E., Green G., Chinchar V.G. (1999). Molecular characterization of iridoviruses isolated from sympatric amphibians and fish. Virus Res..

[47] Moody N., Owens L. (1994). Experimental demonstration of the pathogenicity of frog virus, Bohle iridovirus, for a fish species, barramundi, *Lates calcariger*. Dis. Aquat. Org..

[48] Rodríguez-Meza D., Rodríguez Figueroa G., Sapozhnikov D., Vargas-Ramírez C., Vallejo-Soto A., Verdugo-Quiñonez G., Michel-Rubio A. Monitoreo de la calidad del agua del acuífero de Guasave, Sinaloa Mexico.

[49] Price S.J., Leung W.T.M., Christopher J.O., Sergeant C., Cunningham A.A., Ballous F., Garner T.W.J., Nichols R. A. (2018). Temperature is a key driver of a wildlife epidemic and future warming will increase impact. bioRxiv.

[50] Jancovich J.K., Chinchar V.G., Hyatt A., Miyazaki T., Zhang Q.Y., King A.M.Q., Adams M.J., Carstens E.B., Lefkowitz E. (2012). Virus Taxonomy: Ninth Report of the International Committee of Taxonomy of Viruses.

[51] Saucedo B., Hughes J., Spitzen van der Sluijs A., Kruithof N., Schils M., Rijks J.M., Jacinto-Maldonado M., Suárez N., Haenen O.L.M., Voorbergen-Laarman M. (2018). Ranavirus genotypes in The Netherlands and their potential association with virulence in water frogs (*Pelophylax* spp.). Emerg. Microbes Infect..

[52] Cunningham A.A., Langton T.E.S., Bennet P.W., Lewing J.F., Drury S.E.N., Gough R.E., MacGregor E.K. (1996). Pathological and microbiological findings from incidents of unusual mortality of the common frog (*Rana temporaria*). Philos. Trans. R. Soc. Lond. B Biol. Sci..

