# Supplementary material for: Pathogen Risk Analysis for Wild Amphibian Populations Following the First Report of a Ranavirus Outbreak in Farmed American Bullfrogs (Lithobates catesbeianus) from Northern Mexico
Source: Viruses. 2019 Jan 3;11(1):26. doi: 10.3390/v11010026 (PMC6356443; doi:10.3390/v11010026)
Supplement: Supplementary file 1 [file viruses-11-00026-s001.zip › viruses-375116 - supplementary/Suplementary Table S3. Risk assessment 2.pdf]

Assessment

context

B01. Provide the name(s) of the assessors:
Comments: Virologist and researcher in virus affecting aquatic organisms of commercial interest and wildlife. Instituto Politecnico Nacional-CIIDIR Sinaloa

Weight: None	Answer: unanswered	AValue: Cesar Marcial Escobedo-Bonilla	Confidence: unanswered	CValue: unanswered

B02. Provide the name of the pathogen under assessment:
Comments: A virus pathogen causing disease and mortality to several species of amphibians, reptiles and fish

Weight: None	Answer: unanswered	AValue: Ranavirus FV3	Confidence: unanswered	CValue: unanswered

B03. Provide the name of the host organism under assessment:
Comments: a colony of this frog displayed an outbreak caused by the pathogen producing an unknown number of dead animals

Weight: None	Answer: unanswered	AValue: Lithobates catesbeianus	Confidence: unanswered	CValue: unanswered

B04. Define the area under assessment:
Comments: Outskirts of guasave, a small semi-rural town surrounded by fields used in agriculture of corn, beans, peppers, fruits, flowers, etc. Water canals for irrigation pass in the area

Weight: None	Answer: unanswered	AValue: Northwest Mexico, Northern Sinaloa, Guasave	Confidence: unanswered	CValue: unanswered

B05. This assessment is considering potential impacts within the following domains:
Comments: target species are wild populations of amphibians including frogs, toads, salamanders, some reptiles and freshwater fish

Weight: None	Answer: unanswered	AValue: the environmental domain	Confidence: unanswered	CValue: unanswered

B06. The Pathogen is / would be the cause of a(n) (...) infectious disease to the targets in The Area.
Comments: it is unknown whether wild populations of amphibians harbor the pathogen, but it is possible that the pathogen be re emerging

Weight: None	Answer: (re)emerging	AValue: emerging	Confidence: unanswered	CValue: unanswered

endemic - exposure

B07. Because of The Organism, the probability for The Pathogen to become increasingly prevalent within targets in The Area is:

Weight: None	Answer: medium	AValue: 0.5	Confidence: medium	CValue: 0.5

emerging - entry

B08. The probability of The Pathogen to be introduced with The Organism into The Area is :
Comments: The probability of infected organisms to enter the area carrying the pathogen is considered low assuming that the cultured frogs have a small opportunity to escape from the facility into the surrounding area

Weight: 1	Answer: low	AValue: 0	Confidence: medium	CValue: 0.5

emerging - exposure

B09. The Pathogen has a(n) (...) probability to be maintained and spread within The Organism population in The Area.
Comments: the chance that once the pathogen enters the area, it can be established within a susceptible population is medium.

Weight: 1 Answer: medium AValue: 0.5 Confidence: medium CValue: 0.5

B10. The probability for The Pathogen to be transmitted from individual Organisms to individual targets is:
Comments: the probability of the pathogen to be transmitted from an individual to an individual target is medium, since transmission would depend on the amount of space and time available to produce such an event.

Weight: 1 Answer: medium AValue: 0.5 Confidence: medium CValue: 0.5

environmental

B11. The Pathogen has a (...) effect on native species individuals.
Comments: the pathogen may cause a medium to high effect on native species since susceptible species may show moderate to severe signs of disease and mortality

Weight: 1 Answer: medium AValue: 0.5 Confidence: medium CValue: 0.5

B12. The Pathogen has a (...) effect on native species populations.
Comments: once the pathogen may become established as endemic, the effect on native susceptible populations is considered medium with outbreaks occuring once every 3 years.

Weight: 1 Answer: medium AValue: 0.5 Confidence: medium CValue: 0.5

plant

B13. The Pathogen has a(n) (...) effect on individual plants.
Comments: the pathogen is known to be harmless to plants

Weight: n/a Answer: inapplicable AValue: n/a Confidence: high CValue: 1

B14. The Pathogen has a(n) (...) effect on plant populations
Comments: the pathogen does not affect plants

Weight: n/a Answer: inapplicable AValue: n/a Confidence: high CValue: 1

animal

B15. The Pathogen has a(n) (...) effect on the health (physical well-being and welfare) of individual animals.
Comments: The pathogen does not affect domestic warm-blooded animals.

Weight: n/a Answer: inapplicable AValue: n/a Confidence: high CValue: 1

B16. The Pathogen has a(n) (...) effect on the health (physical well-being and welfare) or production of animal populations.
Comments: The pathogen does not affect domestic warm-blooded animals.

Weight: n/a Answer: inapplicable AValue: n/a Confidence: high CValue: 1

human

B17. The Pathogen has a(n) (...) effect on the health (physical, mental or social well-being) of individual humans.
Comments: The pathogen does not affect humans

Weight: n/a Answer: inapplicable AValue: n/a Confidence: high CValue: 1

B18. The Pathogen has a(n) (...) effect on the health (physical, mental or social well-being) of the human population.
Comments: The pathogen does not affect humans

Weight: n/a Answer: inapplicable AValue: n/a Confidence: high CValue: 1

other

B19. The Pathogen has a(n) (...) effect on international trade and tourism.

Comments: so far the presence of the pathogen in cultured animals has no effect on international trade and/or tourism

Weight: 1	Answer: low	AValue: 0	Confidence: medium	CValue: 0.5

B20. The Pathogen has a(n) (...) effect on public attention and perception.

Comments: so far, the public attention has not been affected by the presence of the pathogen

Weight: 1	Answer: low	AValue: 0	Confidence: medium	CValue: 0.5

Summary

Module	Score	Aggregation method	Weight	Confidence
emerging - entry score	0.0	arithmetic	1	0.5
emerging - exposure score	0.5	arithmetic	1	0.5
environmental score	0.5	arithmetic	1	0.5
plant score	n/a	arithmetic	1	n/a
animal score	n/a	arithmetic	1	n/a
human score	n/a	arithmetic	1	n/a
other score	0.0	arithmetic	1	0.5
Consequence	0.500	maximum		
Entry-Exposure	0.000	geometric		
overall risk score	0.000			
