# Supplementary material for: Pathogen Risk Analysis for Wild Amphibian Populations Following the First Report of a Ranavirus Outbreak in Farmed American Bullfrogs (Lithobates catesbeianus) from Northern Mexico
Source: Viruses. 2019 Jan 3;11(1):26. doi: 10.3390/v11010026 (PMC6356443; doi:10.3390/v11010026)
Supplement: Supplementary file 1 [file viruses-11-00026-s001.zip › viruses-375116 - supplementary/Supplementary Table S1.Comparison of genomic identity between Mexican ranavirus and other ranaviruses..pdf]

Supplementary File S1. Comparison of genomic identity between Mexican ranavirus and other ranaviruses.

ORF13L Hypothetical protein gene, GenBank accession no. MK204475

Ranavirus isolates	Genomic identity	GenBank no.
Frog virus 3	99.64%	AY548484
Frog virus 3 isolate SSME	99.28%	KJ175144
Rana grylio iridovirus	99.64%	JQ654586
Soft-shelled turtle iridovirus	99.64%	EU627010
Frog virus 3 isolate Op/2015/Netherlands/UU3150324001	97.50%	MF360246
Testudo hermanni ranavirus isolate CH8/96	97.50%	KP266741
Chinese giant salamander iridovirus isolate CGSIV-HN1104	97.50%	KF512820
Andrias davidianus ranavirus isolate 2010SX	97.50%	KF033124
Andrias davidianus ranavirus isolate 1201	97.50%	KC865735
Bosca's newt virus isolate GA11001	96.80%	KJ703145
Common midwife toad virus PE11114	96.11%	KJ703147
Common midwife toad virus	95.75%	JQ231222
Tiger frog virus	96.08%	AF389451
Tortoise ranavirus	95.05%	KP266743
Ambystoma tigrinum stebbensi virus	93.59%	AY150217
Epizootic hematopoietic necrosis virus	92.90%	FJ433873
European sheatfish virus	92.90%	JQ724856

ORF22L Proliferating cell nuclear antigen gene , GenBank accession no. MK204476

Ranavirus isolates	Genomic identity	GenBank no.
Frog virus 3	100%	AY548484
Frog virus 3 isolate SSME	100%	KJ175144
Frog virus 3 isolate Op/2015/Netherlands/UU3150324001	99.36%	MF360246
Rana grylio iridovirus	99.36%	JQ654586
Soft-shelled turtle iridovirus	99.36%	EU627010
Testudo hermanni ranavirus isolate CH8/96	98.73%	KP266741
Chinese giant salamander iridovirus isolate CGSIV-HN1104	98.73%	KF512820
Andrias davidianus ranavirus isolate 2010SX	98.73%	KF033124
Andrias davidianus ranavirus isolate 1201	98.73%	KC865735
Common midwife toad virus PE11114	98.73%	KJ703152
Common midwife toad virus	98.73%	JQ231222
European sheatfish virus	98.42%	JQ724856
Bosca's newt virus isolate GA11001	98.10%	KJ703143
Tiger frog virus	98.10%	AF389451
Epizootic hematopoietic necrosis virus	98.10%	FJ433873
Tortoise ranavirus	97.47%	KP266743
Ambystoma tigrinum stebbensi virus	96.52%	AY150217

ORF59R Hypothetical protein gene, GenBank accession no. MK204477

Ranavirus isolates	Genomic identity	GenBank no.
Frog virus 3	100%	AY548484
Frog virus 3 isolate SSME	100%	KJ175144
Frog virus 3 isolate Op/2015/Netherlands/UU3150324001	100%	MF360246
Rana grylio iridovirus	100%	JQ654586
Soft-shelled turtle iridovirus	100%	EU627010
Bosca's newt virus isolate GA11001	99.66%	KJ703139
Testudo hermanni ranavirus isolate CH8/96	99.33%	KP266741
Chinese giant salamander iridovirus isolate CGSIV-HN1104	99.33%	KF512820
Andrias davidianus ranavirus isolate 2010SX	99.33%	KF033124
Andrias davidianus ranavirus isolate 1201	99.33%	KC865735
Common midwife toad virus PE11114	99.33%	KJ703147
Common midwife toad virus	99.33%	JQ231222
Tiger frog virus	99.33%	AF389451
Epizootic hematopoietic necrosis virus	99.33%	FJ433873
Tortoise ranavirus	98.66%	KP266743
European sheatfish virus	98.66%	JQ724856
Ambystoma tigrinum stebbensi virus	96.66%	AY150217

ORF82L p31K gene, GenBank accession no. MK204478

Ranavirus isolates	Genomic identity	GenBank no.
Frog virus 3	100%	AY548484
Frog virus 3 isolate SSME	100%	KJ175144
Frog virus 3 isolate Op/2015/Netherlands/UU3150324001	100%	MF360246
Rana grylio iridovirus	99.07%	JQ654586
Soft-shelled turtle iridovirus	99.07%	EU627010
Testudo hermanni ranavirus isolate CH8/96	99.07%	KP266741
Chinese giant salamander iridovirus isolate CGSIV-HN1104	99.07%	KF512820
Andrias davidianus ranavirus isolate 2010SX	99.07%	KF033124
Andrias davidianus ranavirus isolate 1201	99.07%	KC865735
Common midwife toad virus	98.76%	JQ231222
Common midwife toad virus PE11114	99.38%	KJ703160
Tortoise ranavirus	98.76%	KP266743
Tiger frog virus	98.46%	AF389451
Epizootic hematopoietic necrosis virus	98.46%	FJ433873
Bosca's newt virus isolate GA11001	98.48%	KJ703122
European sheatfish virus	98.15%	JQ724856
Ambystoma tigrinum stebbensi virus	96.92%	AY150217

ORF 16L Major capsid protein gene GenBank accession no. MH247258

Ranavirus isolates	Genomic identity	GenBank no.
Frog virus 3	100%	AY548484
Frog virus 3 isolate SSME	100%	KJ175144
Rana grylio iridovirus	99.78%	JQ654586
Soft-shelled turtle iridovirus	99.78%	EU627010
Frog virus 3 isolate Op/2015/Netherlands/UU3150324001	99.78%	MF360246
Common midwife toad virus PE11114	98.70%	KJ703125
Common midwife toad virus	98.70%	JQ231222
Testudo hermanni ranavirus isolate CH8/96	98.48%	KP266741
Chinese giant salamander iridovirus isolate CGSIV-HN1104	98.48%	KF512820
Andrias davidianus ranavirus isolate 2010SX	98.48%	KF033124
Andrias davidianus ranavirus isolate 1201	98.48%	KC865735
Bosca’s newt virus isolate GA11001	98.48%	KJ703143
Tiger frog virus	98.48%	AF389451
Tortoise ranavirus	98.48%	KP266743
Epizootic hematopoietic necrosis virus	97.83%	FJ433873
European sheatfish virus	97.18%	JQ724856
Ambystoma tigrinum stebbensi virus	95.88%	AY150217
