# Supplementary material for: Pathogen Risk Analysis for Wild Amphibian Populations Following the First Report of a Ranavirus Outbreak in Farmed American Bullfrogs (Lithobates catesbeianus) from Northern Mexico
Source: Viruses. 2019 Jan 3;11(1):26. doi: 10.3390/v11010026 (PMC6356443; doi:10.3390/v11010026)
Supplement: Supplementary file 1 [file viruses-11-00026-s001.zip › viruses-375116 - supplementary/Supplementary Table S2. Risk assessment 1.pdf]

Assessment

context

B01. Provide the name(s) of the assessors:
Comments: Veterinary pathologist

Weight: None	Answer: unanswered	AValue: Bernardo Saucedo	Confidence: unanswered	CValue: unanswered

B02. Provide the name of the pathogen under assessment:
Comments: Frog virus 3

Weight: None	Answer: unanswered	AValue: Ranavirus	Confidence: unanswered	CValue: unanswered

B03. Provide the name of the host organism under assessment:

Weight: None	Answer: unanswered	AValue: Lithobates catesbeianus, American bullfrog	Confidence: unanswered	CValue: unanswered

B04. Define the area under assessment:

Weight: None	Answer: unanswered	AValue: Guasave, Sinaloa, Mexico	Confidence: unanswered	CValue: unanswered

B05. This assessment is considering potential impacts within the following domains:

Weight: None	Answer: unanswered	AValue: the environmental domain	Confidence: unanswered	CValue: unanswered

B06. The Pathogen is / would be the cause of a(n) (...) infectious disease to the targets in The Area.

Weight: None	Answer: (re)emerging	AValue: emerging	Confidence: unanswered	CValue: unanswered

endemic - exposure

B07. Because of The Organism, the probability for The Pathogen to become increasingly prevalent within targets in The Area is:
Comments: Since amphibian species in the area have never been exposed to ranavirus and many of these species have been severely affected around the world, there is a possibility that they are greatly affected and most likely to succumb to infection , prevalence depends on the species abbility to harbor subclinical infections , most likely in relation to immune status or life stage

Weight: None	Answer: medium	AValue: 0.5	Confidence: medium	CValue: 0.5

emerging - entry

B08. The probability of The Pathogen to be introduced with The Organism into The Area is :
Comments: Comments: Given the possibility that farmed bullfrogs escape the affected enclosure and their abbility to disperse for long distance and to harbor subclinical infections, it is likely that these bullfrogs can introduce the disease into wild populations. There is also the possibility that virus-contaminated water reaches natural waterbodies through general sewage system

Weight: 1	Answer: medium	AValue: 0.5	Confidence: medium	CValue: 0.5

emerging - exposure

B09. The Pathogen has a(n) (...) probability to be maintained and spread within The Organism population in The Area.
Comments: The pathogen has been shown to be mantained in waterbodies, reservoir hosts (like bullfrogs), fomites (for example in the feathers of birds) and moist soil.

Weight: 1 Answer: high AValue: 1 Confidence: medium CValue: 0.5

B10. The probability for The Pathogen to be transmitted from individual Organisms to individual targets is:
Comments: The high capacity of Ranavirus for effective transmission not only among co-specifics but also across species is well documented

Weight: 1 Answer: high AValue: 1 Confidence: high CValue: 1

environmental

B11. The Pathogen has a (...) effect on native species individuals.
Comments: The pathogen is able to cause high mortality amongst amphibians, reptiles and fish in the wild, it has the potential to cause extinction and massive economical loss to the fish industry

Weight: 1 Answer: high AValue: 1 Confidence: high CValue: 1

B12. The Pathogen has a (...) effect on native species populations.
Comments: Native susceptible amphibian species in Sinaloa (the province where the affected farm is located) include caudates belonging to the families Ambystomatidae (1 species) and Plethodontidae (1 species), and anurans belonging to Bufonidae toads (9 species) and Scaphiopodidae (1 species) Hylidae (11 species), Leptodactylidae (9 species), Mycrohylidae (3 species) and Ranidae (5 species). They have all shown to be affected by ranaviruses in the past. The chances of the pathogen to become established within the area and causing mortality in these species is high, and animals are likely to experience yearly mortality.

Weight: 1 Answer: high AValue: 0.75 Confidence: high CValue: 1

plant

B13. The Pathogen has a(n) (...) effect on individual plants.
Comments: The pathogen does not affect plants.

Weight: n/a Answer: inapplicable AValue: n/a Confidence: high CValue: 1

B14. The Pathogen has a(n) (...) effect on plant populations
Comments: The pathogen does not affect plants.

Weight: n/a Answer: inapplicable AValue: n/a Confidence: high CValue: 1

animal

B15. The Pathogen has a(n) (...) effect on the health (physical well-being and welfare) of individual animals.
Comments: The pathogen does not affect domestic animals

Weight: n/a Answer: inapplicable AValue: n/a Confidence: high CValue: 1

B16. The Pathogen has a(n) (...) effect on the health (physical well-being and welfare) or production of animal populations.
Comments: The pathogen does not affect domestic animals

Weight: n/a Answer: inapplicable AValue: n/a Confidence: high CValue: 1

human

B17. The Pathogen has a(n) (...) effect on the health (physical, mental or social well-being) of individual humans.
Comments: The pathogen does not affect humans

Weight: n/a Answer: inapplicable AValue: n/a Confidence: high CValue: 1

B18. The Pathogen has a(n) (...) effect on the health (physical, mental or social well-being) of the human population.
Comments: The pathogen does not affect humans

Weight: n/a Answer: inapplicable AValue: n/a Confidence: medium CValue: 0.5

other

B19. The Pathogen has a(n) (...) effect on international trade and tourism.
Comments: Given the abbility to cause mortality on amphibian populations, this can cause severe disruption of the surrounding habitats and increase problems of plagues (mosquitos) making affected areas less appealing to tourists. The bullfrogs are usually only transported to areas within the country, so international trade is not likley to take place in the near future.

Weight: 1 Answer: low AValue: 0 Confidence: medium CValue: 0.5

B20. The Pathogen has a(n) (...) effect on public attention and perception.
Comments: Severe mortality in cold blooded vertebrates is bound to draw public attention due to the concerns for the environment.

Weight: 1 Answer: medium AValue: 0.5 Confidence: medium CValue: 0.5

Summary

Module	Score	Aggregation method	Weight	Confidence
emerging - entry score	0.5	arithmetic	1	0.5
emerging - exposure score	1.0	arithmetic	1	0.75
environmental score	0.875	arithmetic	1	1.0
plant score	n/a	arithmetic	1	n/a
animal score	n/a	arithmetic	1	n/a
human score	n/a	arithmetic	1	n/a
other score	0.25	arithmetic	1	0.5
Consequence	0.875	maximum		
Entry-Exposure	0.707	geometric		
overall risk score	0.619			
