# Supplementary material for: Pathogen Risk Analysis for Wild Amphibian Populations Following the First Report of a Ranavirus Outbreak in Farmed American Bullfrogs (Lithobates catesbeianus) from Northern Mexico
Source: Viruses. 2019 Jan 3;11(1):26. doi: 10.3390/v11010026 (PMC6356443; doi:10.3390/v11010026)
Supplement: Supplementary file 1 [file viruses-11-00026-s001.zip › viruses-375116 - supplementary/Supplementary Table S4. Risk assessment 3.pdf]

Biodiversity.be

Assessment

context

B01. Provide the name(s) of the assessors:
Comments: Biologist

Weight: None	Answer: unanswered	AValue: José Manuel Serrano	Confidence: unanswered	CValue: unanswered

B02. Provide the name of the pathogen under assessment:

Weight: None	Answer: unanswered	AValue: Ranavirus	Confidence: unanswered	CValue: unanswered

B03. Provide the name of the host organism under assessment:

Weight: None	Answer: unanswered	AValue: American bullfrog	Confidence: unanswered	CValue: unanswered

B04. Define the area under assessment:

Weight: None	Answer: unanswered	AValue: Guasave, Sinaloa Mexico	Confidence: unanswered	CValue: unanswered

B05. This assessment is considering potential impacts within the following domains:

Weight: None	Answer: unanswered	AValue: the environmental domain	Confidence: unanswered	CValue: unanswered

B06. The Pathogen is / would be the cause of a(n) (...) infectious disease to the targets in The Area.

Weight: None	Answer: (re)emerging	AValue: emerging	Confidence: unanswered	CValue: unanswered

endemic - exposure

B07. Because of The Organism, the probability for The Pathogen to become increasingly prevalent within targets in The Area is:

Weight: None	Answer: medium	AValue: 0.5	Confidence: medium	CValue: 0.5

emerging - entry

B08. The probability of The Pathogen to be introduced with The Organism into The Area is :

Weight: 1	Answer: high	AValue: 1	Confidence: medium	CValue: 0.5

emerging - exposure

B09. The Pathogen has a(n) (...) probability to be maintained and spread within The Organism population in The Area.

Weight: 1	Answer: medium	AValue: 0.5	Confidence: high	CValue: 1

B10. The probability for The Pathogen to be transmitted from individual Organisms to individual targets is:

Weight: 1	Answer: high	AValue: 1	Confidence: medium	CValue: 0.5

environmental

B11. The Pathogen has a (...) effect on native species individuals.

Weight: 1 Answer: high AValue: 1 Confidence: high CValue: 1

B12. The Pathogen has a (...) effect on native species populations.

Weight: 1 Answer: high AValue: 0.75 Confidence: medium CValue: 0.5

plant

B13. The Pathogen has a(n) (...) effect on individual plants.

Weight: n/a Answer: inapplicable AValue: n/a Confidence: high CValue: 1

B14. The Pathogen has a(n) (...) effect on plant populations

Weight: n/a Answer: inapplicable AValue: n/a Confidence: high CValue: 1

animal

B15. The Pathogen has a(n) (...) effect on the health (physical well-being and welfare) of individual animals.

Weight: n/a Answer: inapplicable AValue: n/a Confidence: high CValue: 1

B16. The Pathogen has a(n) (...) effect on the health (physical well-being and welfare) or production of animal populations.

Weight: n/a Answer: inapplicable AValue: n/a Confidence: high CValue: 1

human

B17. The Pathogen has a(n) (...) effect on the health (physical, mental or social well-being) of individual humans.

Weight: n/a Answer: inapplicable AValue: n/a Confidence: high CValue: 1

B18. The Pathogen has a(n) (...) effect on the health (physical, mental or social well-being) of the human population.

Weight: n/a Answer: inapplicable AValue: n/a Confidence: high CValue: 1

other

B19. The Pathogen has a(n) (...) effect on international trade and tourism.

Weight: 1 Answer: medium AValue: 0.5 Confidence: medium CValue: 0.5

B20. The Pathogen has a(n) (...) effect on public attention and perception.

Weight: 1 Answer: low AValue: 0 Confidence: medium CValue: 0.5

Summary

Module	Score	Aggregation method	Weight	Confidence
emerging - entry score	1.0	arithmetic	1	0.5
emerging - exposure score	0.75	arithmetic	1	0.75
environmental score	0.875	arithmetic	1	0.75
plant score	n/a	arithmetic	1	n/a
animal score	n/a	arithmetic	1	n/a
human score	n/a	arithmetic	1	n/a
other score	0.25	arithmetic	1	0.5
Consequence	0.875	maximum		
Entry-Exposure	0.866	geometric		
overall risk score	0.758			
