# Supplementary material for: Pathogen Risk Analysis for Wild Amphibian Populations Following the First Report of a Ranavirus Outbreak in Farmed American Bullfrogs (Lithobates catesbeianus) from Northern Mexico
Source: Viruses. 2019 Jan 3;11(1):26. doi: 10.3390/v11010026 (PMC6356443; doi:10.3390/v11010026)
Supplement: Supplementary file 1 [file viruses-11-00026-s001.zip › viruses-375116 - supplementary/Supplementary Table S5. Risk asessment 4.pdf]

Assessment

context

B01. Provide the name(s) of the assessors:

Comments: Specialist in ecology and wildlife

Weight: None	Answer: unanswered	AValue: Mónica Jacinto-Maldonado	Confidence: unanswered	CValue: unanswered

B02. Provide the name of the pathogen under assessment:

Weight: None	Answer: unanswered	AValue: Ranavirus	Confidence: unanswered	CValue: unanswered

B03. Provide the name of the host organism under assessment:

Weight: None	Answer: unanswered	AValue: American bullfrog	Confidence: unanswered	CValue: unanswered

B04. Define the area under assessment:

Weight: None	Answer: unanswered	AValue: Guasave Sinaloa	Confidence: unanswered	CValue: unanswered

B05. This assessment is considering potential impacts within the following domains:

Weight: None	Answer: unanswered	AValue: the environmental domain	Confidence: unanswered	CValue: unanswered

B06. The Pathogen is / would be the cause of a(n) (...) infectious disease to the targets in The Area.

Comments: In Guasave there is a large amount of amphibians (37 amphibians in wildlife) which might be at risk

Weight: None	Answer: (re)emerging	AValue: emerging	Confidence: unanswered	CValue: unanswered

endemic - exposure

B07. Because of The Organism, the probability for The Pathogen to become increasingly prevalent within targets in The Area is:

Comments: The probability is medium (moderate) since contaminated water from the farm is not treated prior to disposal through the sewage and virus-contaminated water can infect amphibians from wildlife

Weight: None	Answer: medium	AValue: 0.5	Confidence: medium	CValue: 0.5

emerging - entry

B08. The probability of The Pathogen to be introduced with The Organism into The Area is :

Comments: Very probable since amphibians live in the area that can have contact through the pathogen in water

Weight: 1	Answer: medium	AValue: 0.5	Confidence: medium	CValue: 0.5

emerging - exposure

B09. The Pathogen has a(n) (...) probability to be maintained and spread within The Organism population in The Area.
Comments: Once the pathogen gets access to wildlife it has the possibility to affect susceptible amphibians. Infection is dependent on life stage and adults may act as carriers.

Weight: 1	Answer: high	AValue: 1	Confidence: medium	CValue: 0.5

B10. The probability for The Pathogen to be transmitted from individual Organisms to individual targets is:
Comments: There is a great possibility since the pathogen may access water which may infect amphibian larvae, reptiles and fish

Weight: 1	Answer: high	AValue: 1	Confidence: medium	CValue: 0.5

environmental

B11. The Pathogen has a (...) effect on native species individuals.
Comments: Yes it is very posible that is has an effect since families from these endemic amphibian species have also been affected by ranaviruses

Weight: 1	Answer: high	AValue: 1	Confidence: high	CValue: 1

B12. The Pathogen has a (...) effect on native species populations.

Weight: 1	Answer: high	AValue: 0.75	Confidence: high	CValue: 1

plant

B13. The Pathogen has a(n) (...) effect on individual plants.
Comments: The pathogen does not affect plants

Weight: n/a	Answer: inapplicable	AValue: n/a	Confidence: high	CValue: 1

B14. The Pathogen has a(n) (...) effect on plant populations
Comments: The pathogen does not affect plants

Weight: n/a	Answer: inapplicable	AValue: n/a	Confidence: high	CValue: 1

animal

B15. The Pathogen has a(n) (...) effect on the health (physical well-being and welfare) of individual animals.
Comments: The pathogen only affects cold-blooded vertebrates

Weight: n/a	Answer: inapplicable	AValue: n/a	Confidence: high	CValue: 1

B16. The Pathogen has a(n) (...) effect on the health (physical well-being and welfare) or production of animal populations.
Comments: The pathogen only affects cold-blooded vertebrates

Weight: n/a	Answer: inapplicable	AValue: n/a	Confidence: high	CValue: 1

human

B17. The Pathogen has a(n) (...) effect on the health (physical, mental or social well-being) of individual humans.
Comments: Ranavirus does not affect humans

Weight: n/a	Answer: inapplicable	AValue: n/a	Confidence: high	CValue: 1

B18. The Pathogen has a(n) (...) effect on the health (physical, mental or social well-being) of the human population.
Comments: Ranavirus does not affect humans

Weight: n/a	Answer: inapplicable	AValue: n/a	Confidence: high	CValue: 1

other

B19. The Pathogen has a(n) (...) effect on international trade and tourism.

Weight: 1 Answer: medium AValue: 0.5 Confidence: medium CValue: 0.5

B20. The Pathogen has a(n) (...) effect on public attention and perception.

Weight: 1 Answer: high AValue: 1 Confidence: medium CValue: 0.5

Summary

Module	Score	Aggregation method	Weight	Confidence
emerging - entry score	0.5	arithmetic	1	0.5
emerging - exposure score	1.0	arithmetic	1	0.5
environmental score	0.875	arithmetic	1	1.0
plant score	n/a	arithmetic	1	n/a
animal score	n/a	arithmetic	1	n/a
human score	n/a	arithmetic	1	n/a
other score	0.75	arithmetic	1	0.5
Consequence	0.875	maximum		
Entry-Exposure	0.707	geometric		
overall risk score	0.619			
